# The role of oxidative stress in intervertebral disc cellular senescence

**DOI:** 10.3389/fendo.2022.1038171

**Published:** 2022-12-06

**Authors:** Fengqi Cheng, Honghao Yang, Yunzhong Cheng, Yuzeng Liu, Yong Hai, Yangpu Zhang

**Affiliations:** Department of Orthopedic Surgery, Beijing Chao-Yang Hospital, Capital Medical University, Beijing, China

**Keywords:** oxidative stress, intervertebral disc, cellular senescence, signaling pathway, degenerative disc diseases

## Abstract

With the aggravation of social aging and the increase in work intensity, the prevalence of spinal degenerative diseases caused by intervertebral disc degeneration(IDD)has increased yearly, which has driven a heavy economic burden on patients and society. It is well known that IDD is associated with cell damage and degradation of the extracellular matrix. In recent years, it has been found that IDD is induced by various mechanisms (e.g., genetic, mechanical, and exposure). Increasing evidence shows that oxidative stress is a vital activation mechanism of IDD. Reactive oxygen species (ROS) and reactive nitrogen species (RNS) could regulate matrix metabolism, proinflammatory phenotype, apoptosis, autophagy, and aging of intervertebral disc cells. However, up to now, our understanding of a series of pathophysiological mechanisms of oxidative stress involved in the occurrence, development, and treatment of IDD is still limited. In this review, we discussed the oxidative stress through its mechanisms in accelerating IDD and some antioxidant treatment measures for IDD.

## Introduction

With the aging population, the incidence of spinal degenerative diseases caused by disc degeneration is increasing year by year (1). Degenerative disc diseases, including disc herniation, spinal stenosis, vertebral instability, and other symptoms, can cause severe low back pain and even nerve compression symptoms, resulting in a substantial mental and economic pressure on patients and a heavy burden on the health-care system (2–4). Up to now, the pathogenesis of intervertebral disc degeneration (IDD) is not clear. Studies have found that IDD occurs in various mechanisms (genetic, mechanical, and exposure). Each mechanism exerts *via* a series of pathways leading to the ultimate common outcome of disc cell senescence. There are many therapies for IDD, including therapeutic protein injection, stem cell injection, gene therapy, and tissue engineering, besides the most commonly used surgical treatment. However, there is no effective drug intervention (5).

With the development of molecular biology, epigenetics, and immune technology, people have a more comprehensive and in-depth understanding of the pathogenic factors of IDD. Studies have found that IDD is caused by the senescence of disc cells, and cellular senescence is a process of gradual activation resulting from a variety of initiating factors, among which oxidative stress is an important initiating factor leading to the senescence of disc cells (6). Vitro experiments have demonstrated that the rate of intervertebral disc (IVD) cell senescence is associated with high levels of reactive oxygen species (ROS) and reactive nitrogen species (RNS) production. Still, the specific mechanism of oxidative stress-induced IVD cell senescence still need to be explored (7).

Current researches have focused on identifying the mechanisms by which oxidative stress leads to cell senescence in the IVD. In this review, we focus on the role of oxidative stress in the pathogenesis of IDD and highlight the research advances in the pathways through which oxidative stress leads to the senescence of disc IVD cells and important senescence-related factors. Through studying oxidative stress in the pathogenesis of IDD, we optimize the current therapeutic schemes and try to find new therapeutic interventions with strong specificity, safety, and high efficiency to prevent and treat the disease (5).

## Intervertebral disc

IVD is the largest non-vascularized tissue in the human body. It consists of four parts: central nucleus pulposus (NP), annulus fibrosus (AF), cartilage endplate (EP), and bone endplate (BP). Intervertebral disc cells mainly absorb nutrients by diffusion through the EP, so the cell division of the intervertebral disc is inactive, and the repair ability is poor, which is the potential cause of IDD (8).

The gelatinous NP, located in the center of the intervertebral disc, is rich in the extracellular matrix (ECM) and is composed of water (70-90%), proteoglycans (PGS), and type II collagen (Col). PGS and type II Col play an important role in resisting and alleviating the axial pressure of the spine. With the increase of age, the hardening of the NP is gradually aggravated, and the effect of decompression and cushioning is weakened, which is an important reason why the IVD is vulnerable to external force injury (9).

**Figure 1:**
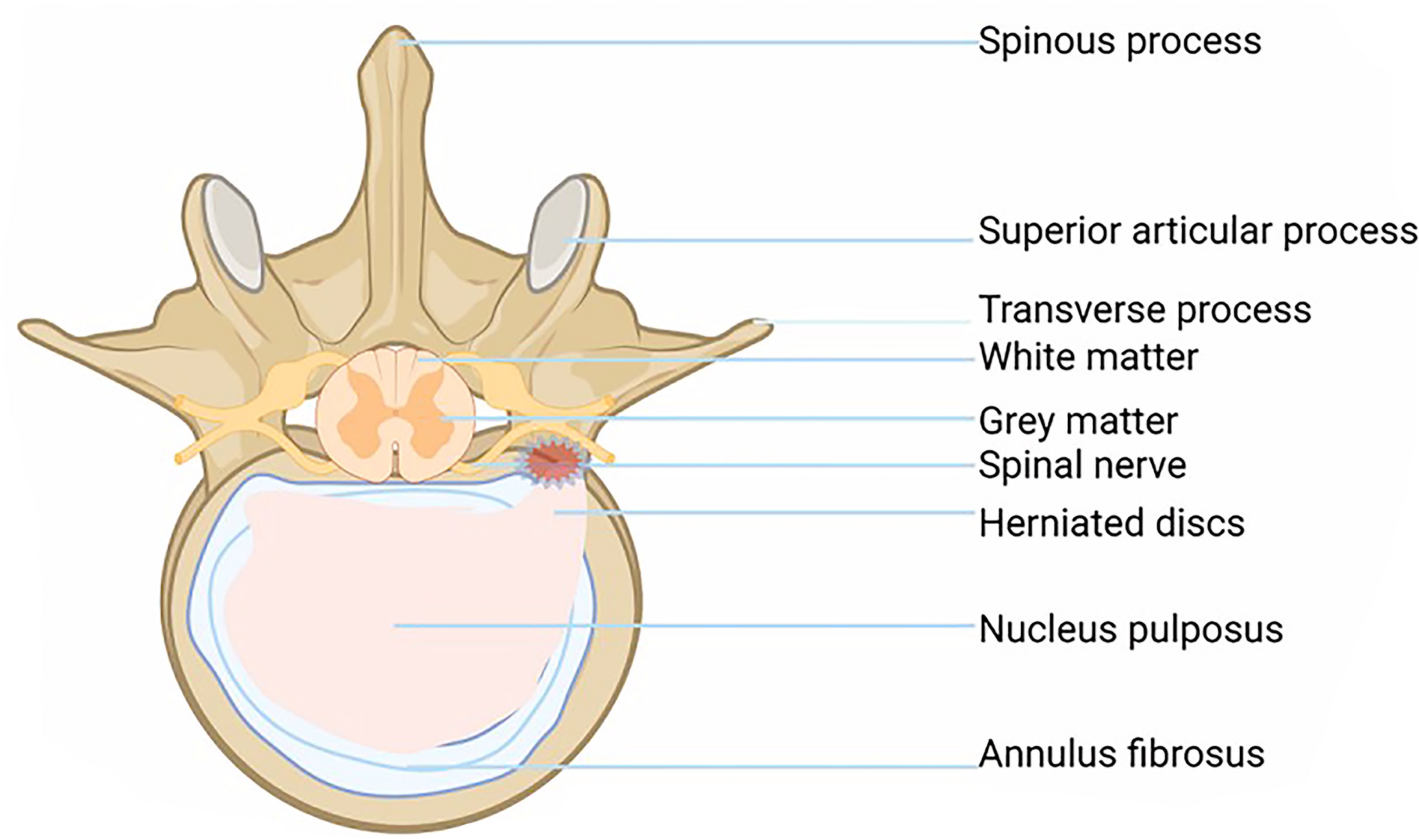
Shows a disc pathology with a horizontal view of a herniated disc and bulging disc above.

The fiber ring is a unique structure composed of 15-25 concentric rings. It looks like a Nylon Mesh pocket and has strong ductility and toughness. The fibrous ring surrounds the NP and bears the pressure from all directions, which plays a protective role in the NP. Each lamina is composed of parallel Col fibers (type I and type II). The fibrous rings are arranged in layers on the cross-section, and the posterolateral part is weak, which is also the reason why IVD herniation tends to occur in the posterolateral part (**Figure 1**) (2, 10).

The endochondral EP is a thin layer of hyaline cartilage, which fixes the IVD on the adjacent vertebrae and acts as a barrier between NP and its upper and lower cancellous bones (11). Endochondral EP cells are distributed in the hyaline cartilage matrix and bind the cartilage disc to the covered vertebrae (12).The cartilage EP is the main contact mode between the IVD and vascular supply: small capillaries penetrate the cartilage EP and make contact with the NP and AP. In the process of IDD, the number of small capillaries decreases, and the bone marrow cavity and cartilage EP calcify, which leads to the reduction of AF and NP blood supply and aggravates the degeneration of the lumbar intervertebral disc (13).

## Intervertebral disc degeneration

During IDD, the degenerative processes at different cellular, molecular, and tissue degeneration levels and their effects on IDD have been a hot topic of research. The different levels of degeneration are causal and contribute to each other, leading to degenerative changes in the intervertebral disc (**Table 1**). For example, IVD cellular senescence is a fundamental cellular program, a multi-linked programmed change influenced by multiple factors involving all aspects of cellular morphology and function. Senescent cells stop responding to mitotic stimuli and undergo significant changes in chromatin structure and gene expression. Cell proliferation, differentiation, and material metabolism are ultimately hindered (14). With the disruption of normal cell structure and function, the secretion of relevant cytokines and degradative enzymes increases, affecting the microenvironment of IVD cell proliferation, differentiation, and material metabolism. These changes further lead to functional and morphological disruption of IVD cells and even apoptosis and autophagy, ultimately leading to IDD development and exacerbation (15, 16).

**Table 1:** Mechanisms and manifestations of intervertebral disc degeneration at different levels.

Level	Degeneration mechanism	Degeneration performance
molecular	DNA is blocked in the g0/g1 phase and could not progress to the next phase.	Cells lose the ability to respond to mitogens and synthesize DNA.
	Abnormal accumulation of inflammation-related factors in the intervertebral disc, such as TNF, IL, NO and leukotrienes.	Inflammatory response and injury.
cellular	Decreased production of extracellular matrix components and increased expression of degradative enzymes	Decrease in extracellular matrix levels.
	Decrease in telomerase activity	The telomere length is gradually shortened.
	Decreased proliferation of articular chondrocytes.	(1) Destruction and reduction of articular cartilage occurs. (2) The lumbar intervertebral disc loses its ability to repair.
organizational	Calcification of cartilage plates.	Decreased exchange of nutrients and metabolites in IVD
	Vascular infiltration can be observed in degenerative disc tissue.	(1) Lymphocytes and macrophages are distributed around the neovascularization, inducing an inflammatory response. (2) Neovascularization delivers more oxygen to degenerated tissues, thus exacerbating oxidative stress.

At the cellular level, apoptosis and autophagy play a key role in IDD. There are two types of programmed cell death, apoptosis, and autophagy (17). Apoptosis occurs in IVD cells in response to oxidative stress, abnormal mechanical stress, and inflammatory factors. Apoptosis occurs through the mitochondrial pathway, the death receptor pathway, and the endoplasmic reticulum pathway (18).When apoptosis occurs, important intracellular organelles and contents are destroyed and degraded, such as causing damage to mitochondria (19).Therefore, apoptosis is an essential factor in oxidative stress leading to degenerative changes in the IVD.

There is a close relationship between cellular autophagy and apoptosis. Autophagy is a protective mechanism of the cell itself. The activation pathways of cellular autophagy include the Mammalian Target of Rapamycin (mTOR)-dependent pathway and the non mTOR-dependent pathway. mTOR-dependent ways include the AMPK/mTOR pathway and the PI3K/Akt/mTOR pathway (20).It was found that basal levels of cellular autophagy were present in normal rat NP cells. However, cellular autophagy levels were significantly increased or decreased in NP tissues that underwent degenerative lesions. Low levels of autophagy induced by mild oxidative stress can inhibit apoptosis and have a protective effect on the cells. Severe oxidative stress or abnormal mechanical stress, resulting in high cellular autophagy levels, caused organelle damage and led to increased apoptosis in myeloid cells (21). In IDD caused by oxidative stress, cellular autophagy plays a different role depends on the degree of disc degeneration changes (22).

In the process of IDD, an imbalance between the anabolic and catabolic metabolism of IVD cells due to a combination of abnormal factors causes the loss of tissue repair capacity, resulting in IDD (23). For example, the ability of articular chondrocytes to proliferate and synthesize decreases, but matrix-degrading enzymes in the cells retain high activity, leading to the destruction and loss of articular cartilage. In addition, the reduction of collagen (Col) and PGS in IVD is an essential indicator of IDD. The synthesis and quality of Col and PGS are also decreased in IVD cells (24, 25).

In addition, protease is also involved in the senescence of IVD cells. As a marker enzyme of cell aging, the positive rate of senescence-associated b-galactosidase (SA-b-Gal). SA-b-Gal increases with the severity of IDD. The results showed that the number of SA-b-Gal positive cells in IVD herniation tissue increased significantly, and the positive rate accounted for 8.5% of the total cells (26). In degenerative IVD, the positive rate of SA-b-Gal in NP cells is higher than that in AP cells, and the proportion of positive cells is as high as 25.5%. This indicates that the aging degree of NP cells in the degenerated IVD is more serious than that in the AP (27).

At the same time, the study found that in degenerated human IVD, not only the expression of SA-b-Gal increased, but also telomerase activity decreased and telomere length shortened (28). Telomere length determines the ability of cell division and proliferation to a certain extent, and telomere length is maintained by telomerase. With the decrease in telomerase activity, the telomere length of cells gradually shortens, and the cell senescence and even apoptosis would occur (29, 30).

Due to the decrease in the production of matrix components and the increase in the expression of degradation enzymes, the dynamic balance between the synthesis and degradation of ECM is disturbed. The reduction of the ECM level of the IVD is a characteristic of IDD. The abnormally high levels of many enzymes, such as matrix metalloproteinase, lectin, and cathepsin, accelerate the catabolism of the ECM. Among them, matrix metalloproteinase-3 (MMP-3) and matrix metalloproteinase-13 (MMP-13) are the key enzymes of ECM degradation in IVD, and interleukin (IL) and tumor necrosis factor (TNF) are important regulators. *In vitro* experiments showed that the activities of MMP-3 and MMP-13 increased significantly in aging IVD (31, 32).

Because the IVD is an avascular tissue, the EP is the key nutritional pathway, and nutrients nourish the NP and AP through the cartilage plate by diffusion. Calcification of the EP reduces the exchange of nutrients and metabolites in IVD, thereby inhibiting the production of an ECM. The imbalance between anabolism and catabolism in the ECM of the IVD leads to the decline of the ECM, thereby accelerating the IDD (33).

At the tissue level, calcification of EP and vascular infiltration interfere with the usual exchange of nutrients and metabolites in the IVD, thereby inhibiting cell proliferation, differentiation, and ECM production. Since the IVD is an avascular tissue, EP is a critical nutrient pathway. Nutrients nourish the NP and AP by diffusion through the cartilage plate, and calcification of the EP disrupts the normal pathway of material exchange (33). In addition, vascular infiltration is an essential factor in promoting IDD. In normal adult IVD, there is no angiogenesis (34). However, vascular infiltration can be observed in degenerative disc tissue. Most neovascularization is located at the edge of the disc and is formed by a single or a few endothelial cells with a very narrow lumen. Vascular endothelial growth factor and p53 may be jointly involved in the process of neo-capillary formation. Coordinated expression of VEGF and p53 was demonstrated in degenerated IVD tissue. In rat degenerative IVD tissues with capillary infiltration, the positive expression of VEGF and p53 was higher than that in the non-infiltrated group. The experimental results suggest that both VEGF and p53 are involved in the process of neovascular infiltration, which accelerates the degeneration of rat IVD tissues (35). Microscopically, lymphocytes and macrophages were distributed around the neovascularization, which could induce an inflammatory response and promote IDD (36).In addition, neovascularization in degenerated discs delivers more oxygen to the degenerated tissue, thus exacerbating the damage to the discs from oxidative stress.

At the molecular level, it was found that the DNA of aging cells was blocked in G0/G1 phase and could not enter the next stage. Cells lose the ability to react with mitogen and synthesize DNA. The content of DNA in cells cannot alter with the normal periodic changes of cells (37). In addition, in the early stage of IDD, due to inflammatory injury, abnormal accumulation of inflammatory-related factors occurred in the IVD, such as TNF, IL, NO, and leukotriene. Among them, IL-8 not only accelerates the degeneration of IVD but also leads to the neuroinflammation infiltration of the nerve root, resulting in low back pain (38–40).

## Modeling of intervertebral disc degeneration

An appropriate model of IDD is the most important step for subsequent mechanism research. The production of animal models of IDD can be broadly divided into natural-induced models, genetic technology-induced models, physical-induced models, chemical-induced models, and biomechanical-induced models.

## Natural-induced models

The spontaneous model refers to the selection of special breeds of animals, such as rhesus monkeys, dogs, and baboons, without human interference, which can naturally develop IDD similar to humans during the growth and development of the experimental animal.

### Genetic technology-induced models

Gene therapy models are designed to induce IDD by genetically suppressing or promoting the expression of genes in experimental animals, resulting in an imbalance between IVD cell anabolism and catabolism. For example, knocking out the Chsy3 gene in mice induces the development of IDD models. The experimental conditions for gene technology are demanding, complex, and limited in studying specific genes (41).

### Physical-induced models

Direct surgical incision of the fibrous ring can form a model of severe IDD, but it is so destructive to the fibrous ring that it is rarely used. Microscopic removal of the NP of the IVD in mice using a tiny scalpel can also successfully create a model of IDD. Alternatively, the NP, the lamina, and the AF can be damaged by puncture (42). Shi et al. successfully established an IDD model by puncturing the rabbit AF with a thicker needle and extracting the NP under a microscope. The IDD model was also induced by puncturing the cartilage EP and blocking the regular nutrient supply by injecting drugs or bone cement (43). Puncture-induced method is the most commonly used method due to the short model construction period and less damage to the spine of experimental animals.

### Chemical-induced models

D-galactose can be injected into the circulatory system of experimental animals, which leads to abnormal metabolism of the whole body, and then leads to structural and functional damage of various organs. The IVD structure and function of the experimental animals will also change, leading to apoptosis of the IVD cells and finally obtaining a IDD model (44). This systemic chemical induction method is highly damaging to the experimental animals, and the degeneration of the systemic system interferes with the experimental results, which ultimately affects the accuracy and reliability of the experiment.

In addition, chemicals such as Papain Chymotrypsin(PC)and Chondroitinase ABC can be injected into the IVD, forming an IDD in the injected segment (45). Experimental studies showed that PC and Chondroitinase ABC were injected into the IVD of rabbits and goats, respectively, and the degree of IDD was exacerbated with increasing doses. Moreover, the proteoglycan and type II collagen expression levels in IVD were much lower than those in the control group (46). The local chemical induction method has few systemic side effects on experimental animals and no apparent interference with the experimental results. However, the quality of injected chemicals is not easy to control, and the puncture sites are prone to infection and bone destruction.

### Biomechanical model

The normal stresses on the disc are altered by the mechanical application of force, unusual behavior, and intervertebral fusion to induce the development of degenerative changes in the IVD. The current compression tests performed in the rat and goat lumbar spine are very effective in constructing degenerative lumbar spine models. The method is feasible and less damaging to the experimental animals; however, it is prone to local infection and spinal cord injury when external forces are applied. Disruption of the connection between the IVDs can impact the balance of forces in the spine and thus alter the normal intervertebral stresses. For example, the removal of the lumbar interspinous ligaments or synovial joints can alter the static balance, and stripping the sacrospinous or severing the erector spinal muscles can alter the dynamic balance (47–49). Special behavioral models, such as the construction of a double hind limb upright rat model, in which young rats have their forelimbs amputated and are forced to stand and walk using both hind limbs or are placed in an upright tube for 4-12 weeks (50). Dissection of the vertebrae of the experimental animals showed that the degeneration of the lumbar spine was very obvious, the height of the intervertebral space and the proteoglycan of the NP were significantly reduced, the laminar arrangement of the fibrous ring was disturbed, and the EP was fractured. The particular behavioral pattern-induced method could resemble the human lumbar spine stresses more closely, but the strategy is ethically controversial and only applicable to small animals.

### Oxidative stress and IDD

Oxidative stress is caused by the imbalance between the production of free radicals and active metabolites (known as oxidants or ROS) and the elimination of this protective mechanism of antioxidants (51). ROS is a series of incompletely reduced oxidation molecules, which generally contain oxygen free radicals, such as superoxide anion (O2-), hydrogen peroxide (H2O2), hydroxyl radical (OH-), and hypochlorite ion (OCL-), with high chemical activity and a relatively short half-life. Highly active oxidation molecules always have harmful effects on living cells, and mitochondria are the main place for the production of oxygen free radicals. In addition to ROS, RNS are also considered to be members of the ROS family, such as nitric oxide (NO) and its oxidized derivatives such as N0_2_, N_2_O_3_, and GSNO, etc. Their chemical properties are as active as ROS, and they can also be used as key regulatory signals during various cell reactions (52).

Relatively excessive accumulation of ROS will lead to mitochondrial dysfunction and the destruction of cellular homeostasis through the oxidative stress pathway. At the same time, oxidative stress can promote the formation of autophagy through transcriptional regulation and post-transcriptional regulation. These include the following molecular signaling pathways, such as ROS-FOXO3-LC3/BNIP3-autophagy, ROS-NRF2-P62-autophagy, ROS-HIF1-BNIP3/NIX-Autophagy, and ROS-Tigar- Autophagy (53).

The levels of oxidative stress biomarkers, including NO, superoxide dismutase (SOD), malondialdehyde (MDA), and advanced oxidative protein products (AOPPs) in the IVD of Wistar rats were evaluated by histological analysis. The experiment showed that with the increase of age, the concentration of NO in IVD significantly increased, and the levels of MDA and AOPP in serum and IVD gradually increased. However, with the increase of age, the SOD activity of serum and IVD gradually decreased. Experiments show that with age, the production of ROS in IVD is excessive, and antioxidants are reduced, indicating that oxidative stress is related to the degenerative changes of IVD (54).

Oxidative stress plays a key role in the occurrence and development of IDD. Oxidative stress destroys DNA, lipids, protein, and ECM in IVD cells through metabolic regulation mechanisms and inflammatory pathways, thereby damaging the microenvironment balance and mechanical function of IVD. For example, increased NO production causes DNA strand breaks and base modifications through the IL-1 pathway, resulting in increased DNA damage (55).

It also promotes apoptosis and autophagy through a variety of mechanisms, reducing the number of living and functional cells in the IVD micro-environment. Studies have shown that oxidative stress increases the apoptosis and subsequent calcification of cartilage EP cells through Ros/P38/Erk/P65 pathway, thereby reducing the nutritional supply to the IVD and accelerating the degeneration of the IVD (56).

In conclusion, oxidative stress participates in the occurrence and development of IDD through a series of pathophysiological mechanisms.

## Oxidative stress signaling pathway

### Micro-RNA signaling pathway

Micro-RNA is an endogenous non-transcriptional micro-RNA, which is produced by DNA transcription and plays an important regulatory role in the process of transcription and translation after gene expression. Micro-RNA can directly bind to the target mRNA, reducing the expression of the protein encoded by the mRNA. In addition, Micro-RNA can also be used as a specific sequence guide of functional Ribonucleo-protein (RNP) to the target RNA so as to change the secondary structure of mRNA and regulate its translation level. Micro-RNA plays an important role in immune regulation, cell differentiation, and proliferation (57, 58).

The MiR-106b family in Micro-RNA is associated with gene expression that regulates the cell cycle. P21 is the direct target of MiR-106b, and p21 silencing plays a key role in the cell cycle phenotype induced by MiR-106b. In addition, MiR-106b covers the DNA damage checkpoint induced by Adriamycin, thereby promoting cell proliferation. Oxidative stress can reduce the expression level of MiR-106b and promote the aging and death of IVD cells. Experiments have proved that in human fibroblasts and trabecular meshwork cells cultured *in vitro*, oxidative stress can lead to the continuous reduction of MiR-106b, thus promoting the expression of the p21 gene, revealing DNA damage checkpoints, thereby blocking the cell cycle and promoting cell death (59).

Additionally, multiple mi-RNA families pass through the p53 pathway, causing the arrest of the cell cycle. P53 is a transcriptional regulator that can regulate the levels of micro-RNA (mi-RNA). MicroRNA-34a (miR-34a) is a direct transcriptional target of p53 in promoting apoptosis. It was experimentally demonstrated that p53 could induce miR-34a expression in cultured cells, and increased expression levels of miR-34a can mildly increase cell apoptosis (60).Under the induction of p53 activation, the expression levels of miR-192 and miR-215 were also increased. MiR-192 and MiR-215 target transcripts of the G (1) and G (2) checkpoints as direct targets, causing the arrest of the cell cycle and leading to apoptosis through regulatory actions between these genes by miRNAs (61).

### NF-κB signaling pathway

NF-κB regulates the expression of many genes, such as genes related to immunity, inflammation, and cell cycle. NF-κB and IK-Bs binding exists in the cytoplasm as an inactive form. NF-κB is rapidly activated by cytokines, infectious factors, and oxidative factors (e.g., ROS) (62). For example, ROS can activate IK-Bs Kinase (IKK) through ROS-Hsp27-Ikkbeta mediated signal pathway, promoting phosphorylation of IK-Bs, and then IK-Bs is recognized by ubiquitinase and degraded, releasing NF-κB bound by IK-Bs and activating NF-κB signaling pathway (63).

The activation of NF- κB through the NF- κB/p65 pathway promotes the expression of the downstream signal molecule–p65 in the nucleus (64). Experiments showed that compared with normal NP cells, NP cells isolated from IDD patients contained less cytoplasm, and the expression level of p65 in the cytoplasm was higher than that in the nucleus of healthy NP cells treated with PDTC. In addition, the level of p65 increased with the prolonged exposure of cells to peroxynitrite (65, 66).NF-κB/p65 pathway can change the expression levels of mRNA or protein of key transcription factors (such as c-Fos and NFATc1) in osteoclast differentiation, leading to the senescence of NP cells. For example, TCF7L2 is expressed in the nucleus of NP cells and promotes the degradation of cell-matrix and the senescence of intervertebral disc cells through p65/NF-κB signaling pathway (67).

In addition, the activation of the NF-κB/p53 signaling pathway is another pathway of disc cellular senescence. Studies have shown that compared with normal NP cells, the expression of p65 and p53 in IDD cells is significantly increased. Moreover, the expression level of p53 was significantly reduced after the transfection of Sip53 (65).In addition, TNF- α can induce apoptosis of NP cells. Experiments show that TNF-α can increase the expression of TRIM14 and the activity of NF-κB/P65, thereby reducing the viability of human NP cells and inducing apoptosis (68). The activation of NF- κB can also promote the release of proteolytic enzymes such as MMPs and ADAMTs, accelerate the degradation of ECM and aggravate IDD by triggering transcription and translation (69, 70).

PHD2 forms a regulatory loop with TNF-α *via* NF-κB, thereby enhancing its own response to the activation of TNF-α. In the aging NP cells, the expression of both PHD2 and catabolic markers induced by TNF-α increase, accelerating the senescence of IVD cells (71).

**Figure 2:**
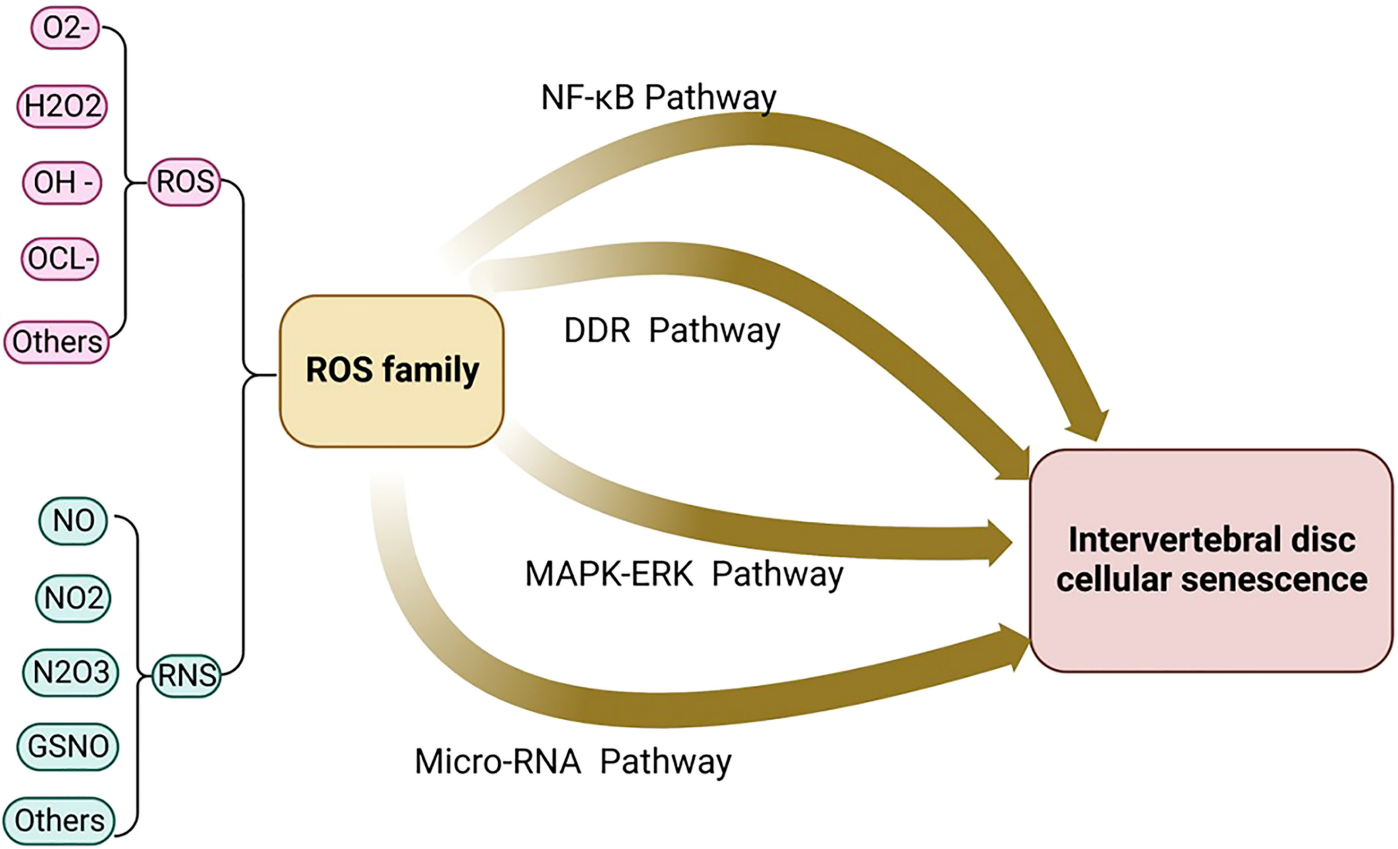
The oxidative stress signaling pathway includes the four major signaling pathways: micro-RNA signaling pathway, NF-kB signaling pathway, MAPK-ERK signaling pathway, and DDR signaling pathway.

**Figure 3:**
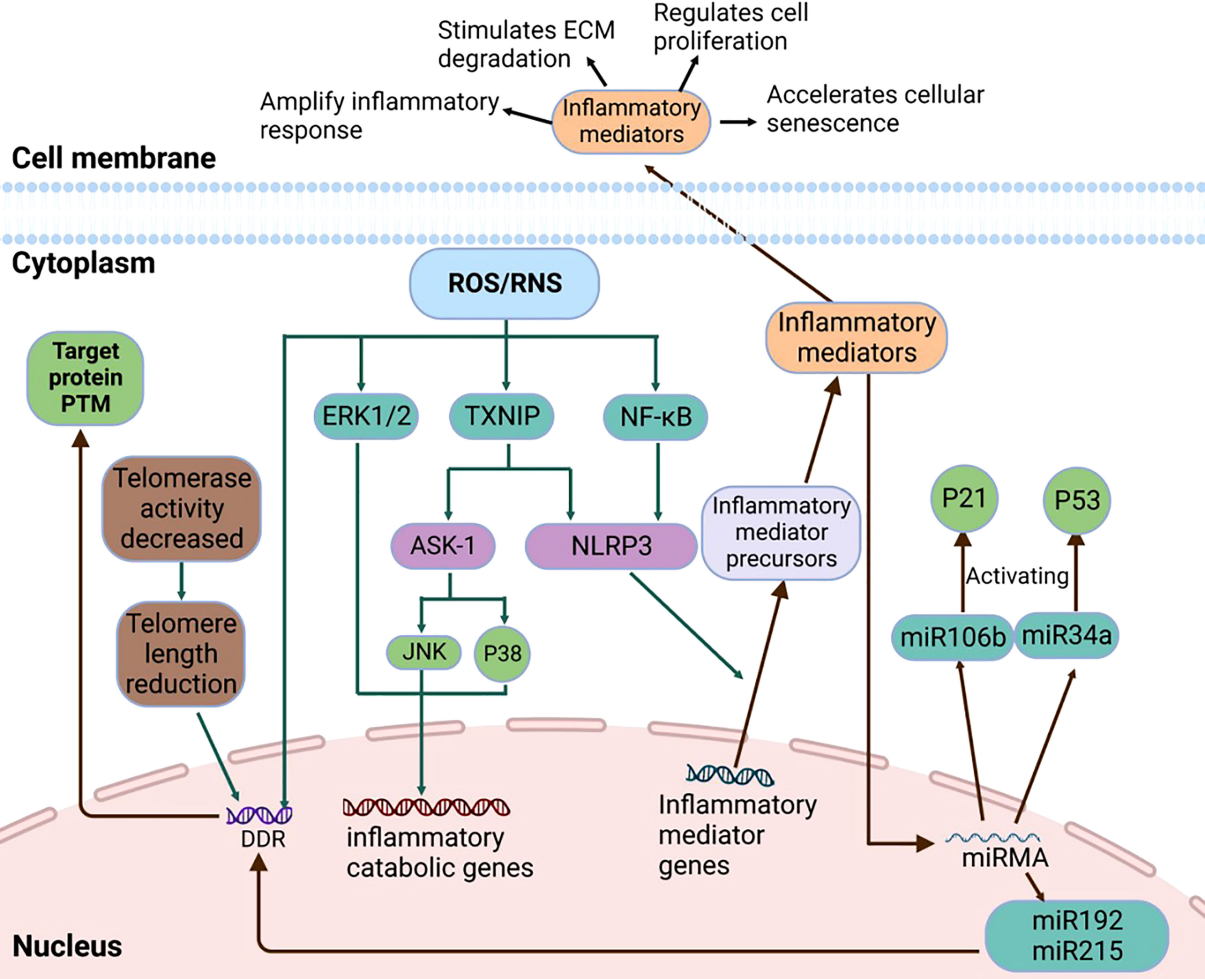
The details of Micro-RNA Signaling Pathway, NF-κB signaling pathway, MAPK signaling pathway, and DDR signaling pathway.

ROS activates NLRP3 in NP cells and promotes the release of intracellular inflammatory factors *via* the NF-κB pathway. For example, the NF-κB signaling pathway stimulates the expression of IL-8, thereby promoting cell aging and apoptosis (63). Research shows that Piezo1 passes the Ca^2+^/NF-κB pathway and activates NLRP3, thereby accelerating the production and maturation of IL-1β and promoting the senescence of IVD (72). The oxidative stress signaling pathways were illustrated in **Figures 2**, **3**.

### MAPK-ERK signaling pathway

Mitogen-activated protein kinases (MAPK) are conserved serine/threonine protein kinases. It also plays an important role in the proliferation, differentiation, and apoptosis of eukaryotic cells. MAPK pathway includes three main kinases: MAPK Kinase, MAPK Kinase, and mitogen-activated protein kinase. They are activated step by step and phosphorylate downstream molecules to complete the transmission of a variety of signals. MAPK can trigger the adaptive response of cells according to the changes in physical and chemical levels of cells. MAPK can regulate the levels and functions of many proteins, including pro-inflammatory factors and factors in p21/p53 and p16/RB pathways so that cells are in a state of infinite growth arrest (73).

Extracellular regulated kinase 1/2 (ERK1/2) is the main type of MAPK kinase. ERK1/2 is essential to maintain the normal physiological function of cells and constitutes a key signaling pathway in various stress-induced apoptosis. Apoptosis signal-regulated kinase 1 (ASK1) is a member of the mitogen-activated protein kinase family. It activates ERK1/2 through MKK4/MKK7-JNK and MKK3/MKK6-p38 MAPKs pathways. ROS/RNS triggers the oxidation of TRX interacting proteins, activates JNK and p38MAPK pathways through the polymerization of ASK, and finally activates the ERK1/2 pathway, resulting in the continuous activation of ERK1/2 (74).Experiments have confirmed that ROS can activate the p38MAPK pathway to regulate the expression of p16 and pl9ARF, limit the self-renewal ability of mouse hematopoietic stem cells, and thus affect the life span of mice (75).

The increased expression of pro-inflammatory cytokines is an important pathological feature of IVD cellular senescence. Endoplasmic reticulum stress regulates the release of inflammatory factors through p38 MAPKs and CHOP pathways. Studies have shown that endoplasmic reticulum stress inducer-thapsigargin (TG) induces the phosphorylation of p38 MAPKs, and p38 MAPKs signal pathway transmits extracellular signals to the nucleus through three-stage cascade amplification, thereby activating the gene expression of IL-6 (76, 77).H_2_O_2_ stimulates autophagy of the IVD through ERK/mTOR signaling pathway and the AMPK/mTOR signaling pathway. In addition, the AMPK/mTOR pathway could also induce neuroinflammation, causing low back pain (78, 79).

Mitogen-activated protein kinase activated protein kinase (Mapkapk) is a protein composed of hundreds of amino acids. It exists uniformly in the cytoplasm and nucleus and can be phosphorylated by ERK and P38MAPKs. Mapkapk5 and PRKCZ pathways have been considered to have the ability to regulate multiple catabolism and are associated with oxidative stress-induced gene methylation (80, 81).

Integrin-β3 is significantly upregulated with cell aging, and p38 MAPK, a key downstream molecule in the senescence pathway mediated by Integrin-β3, is hyperactivated in senescent cells. Retinoic acid-inducible gene-I (RIG-I) is a cytosolic pattern recognition receptor that can alleviate cell senescence, so Rig-I-deficient cells can cause cell senescence *via* integrinβ3/p38 MAPK signal pathway. Studies have shown that compared with wild-type mice, Rig-I-deficient mice have faster hair shedding, and embryonic fibroblasts (MEFs) are more prone to continuous passage related to replicative senescence. Therefore, the p38 MAPK signaling pathway, as a key regulatory pathway of cell senescence, is a regulator of the expression of integrinβ3 in Rig-I-deficient cells (82).

Senescent cells can secrete inflammatory factors, proteases, and transcription factors, known as senescence-related secretory phenotype (SASP) (83).Studies have shown that the expression level of p38 MAPK is upregulated in senescent cells, and p38 MAPK increases NF-κB transcriptional activity and induces SASP. P38 MAPK promoting the SASP pathway is independent of DNA damage response. P53 can inhibit p38 MAPK activation and SASP expression, alleviating apoptosis (84).

### DNA damage response

DNA damage response (DDR) is a signaling pathway that originates from DNA damage and prevents cell proliferation. DNA damage caused by ROS plays an important role in inducing cellular senescence. Nucleotides in DNA molecules are the key targets of ROS. ROS can rapidly induce cell senescence through DDR signaling pathway. In severe cases, the most serious type of DNA damage-DNA double-strand break (DSB) can be formed (85).The DDR pathway is composed of a complex regulatory network, and DDR transmits signals to downstream effector molecules through post-translational modification (PTM) of target proteins and their interactions. Post-translational modifications to target proteins include phosphorylation, ubiquitination, sumoylation, and acetylation, of which phosphorylation is the most common PTM that regulates DDR (86).

In addition, small non-coding RNAs(nc-RNAs) also play an important role in the DDR pathway. miRNAs associated with inflammation and aging (miR-146, miR-155, and miR-21), participating in the DDR pathway through the regulation of nc-RNAs gene expression. Nc-RNAs are processed by Dicer and Drosha RNases to generate small RNAs. Small RNAs produce RNA interference in a Mre11-Rad50-NBS1 complex-dependent manner, causing DNA damage. MiRNA can affect the expression of enzyme proteins and inflammatory factors, and link the inflammatory response with the DDR (87, 88).

The shortening of telomere length is a key signal to trigger the DDR pathway. Since most normal cells cannot fully replicate the linear genome, and the length of chromosome telomeres in cells gradually shortens with cell division (89). When the telomere length is shortened to a certain extent, the DDR pathway is triggered to activate the DNA damage checkpoint kinases CHK1 and CHK2, causing cell cycle arrest (90).

Oxidative stress can cause DSB and other DNA damage. Ataxia telangiectasia mutant kinase (ATM) is a protein kinase induced by DNA damage. ATM is a major regulator of stress response after DSB. It can promote the phosphorylation of DNA damage reaction substrate, up-regulate the activity of p53/p21, stimulate the p19ARF/p53/p21 pathway, and trigger cell senescence (91, 92).

## Cell senescence related factors

### P16 and cell senescence

P16 is a cell cycle inhibitor associated with cellular senescence. INK4a, the gene encoding p16, is located on chromosome 9p21. The p16 protein was able to promote the binding of Rb protein to E2F, thereby inhibiting the phosphorylation of Rb protein. In addition, E2F binding to the Rb protein fails to stimulate the expression of genes related to cell proliferation downstream. Activation of the p16/RB signaling pathway, which can arrest the cell cycle at the G1/S checkpoint, leads to the inhibition of normal cell proliferation or induction of apoptosis (93, 94).

Expression levels of p16 within the cell are regulated by multiple factors. MiRNAs can regulate the transcription of p16. In mouse MEFs, a tight link exists between genes associated with MEF senescence (p19ARF, p16, p21) and the miRNA family (miR-20a, miR-21, mir-28, miR-290). For example, miR-20a can upregulate the transcriptional activity of INK4a/ARF, leading to cell senescence (95, 96). In addition, acetylation of intracellular proteins can elevate the expression of p16. For example, the acetylation modification of histones can promote the activity of INK4A, activating the expression of p16. Acetylation of the transcription factor HBP1 by p300/CBP, which also enhances the expression of p16, accelerates cellular senescence (97, 98).

Loss and methylation of the promoter in the gene encoding INK4a can inhibit the expression of p16, thereby delaying cellular senescence (99). A-U-rich elements at the 3’ untranscribed end can bind to the 3’ untranscribed end of the mRNA of p16, and the binding of AUF1 to the 3’UTR of p16 promotes the degradation of p16 and has a significant anti-aging effect. Experimentally, HeLa cells overexpressing AUF1 are resistant to cellular senescence induced by H_2_O_2_ (100).

### ARF/P53/P21 and cell senescence

**Figure 4:**
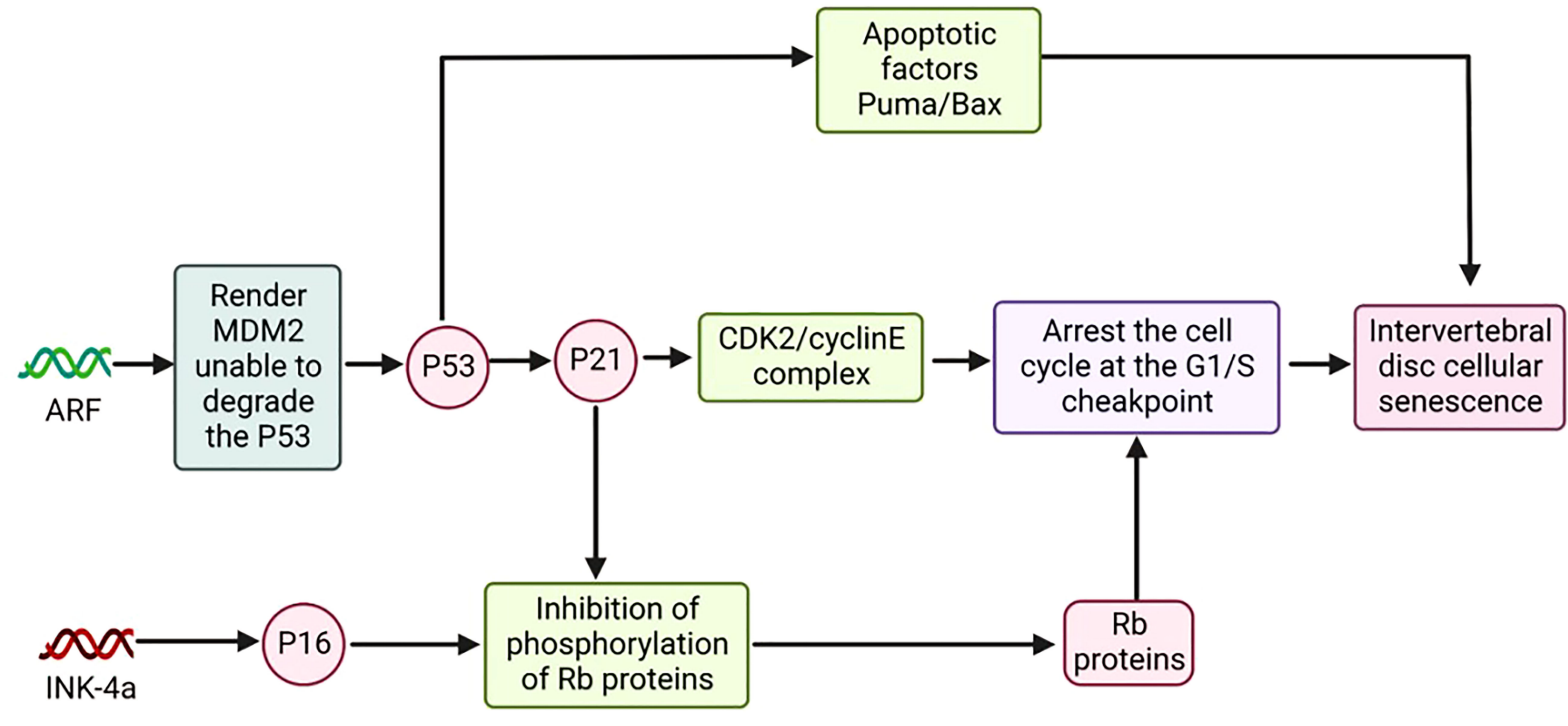
The Model of P16 and Cell Senescence and ARF/P53/P21 and Cell Senescence.

P53 encoded by the p53 gene is a tumor suppressor protein; P21 encoded by the p21 gene is a cell cycle-associated inhibitory protein (101, 102). ARF regulates the expression of p53 and p21, and the gene encoding ARF is located on chromosome 9p21. The ARF/p53/p21 pathway is an important regulatory pathway of cellular senescence (**Figure 4**) (103).

Compared with the p16 pathway, the ARF/p53/p21 pathway is relatively complex. P53 can cause cell senescence by activating p21, and in addition, p53 itself promotes the expression of apoptotic factors Puma and Bax, among others, to induce apoptosis (104).ARF renders MDM2 unable to bind E3 by binding to the ubiquitin ligase MDM2. MDM2, which is not bound to E3, has lost the ability to degrade the p53 protein, thereby increasing the expression level of p53 in cells and promoting the expression of p21. P21 can inhibit the activity of the CDK2/cyclinE complex and keep pRb in a hypophosphorylated or dephosphorylated state. The inactive state of PRB can bind to E2F and arrest the normal cell cycle (105, 106).

The expression level of p53 in cells is regulated by multiple pathways. Damaged DNA in senescent cells induces the expression of p53 through the ATM pathway (107).In response to DNA damage and oxidative stress, the acetylation of p53 is also essential for regulating the transcriptional activity of p53 (108).As a multifunctional phosphoprotein, Artemis is also a key protein of non-homologous terminal junction (NHEJ). Under oxidative stress, Artemis and DNA PKC participate in the negative regulation of p53 (109). P19ARF/p53/p21 is one of the major signaling pathways of senescence responses in cells. When DNA is damaged, p53 can activate the expression of a variety of related genes to arrest the cell cycle in G1 and G2 phases, thereby preventing cell proliferation and inducing cell senescence (110).

Experiments have shown that elevating the expression levels of p53 or p21 in cells can induce a cellular senescence response. The expression levels of ARF, p53, and p21 were elevated in senescent MEFs cells and during senescence induced by the oncogenic signal H-ras. Mouse fibroblasts with humanized p53 (Hupki cells, derived from a human p53 knock-in mouse model) first senesce (111, 112).In addition, telomeres become progressively shorter in length, and once the length reaches a critical threshold, the telomeres signal to p53 for growth arrest *via* the ATM pathway, and upregulate p21 to lead to G1 arrest *via* the p19ARF/p53/p21 pathway, ultimately leading to cell senescence (113, 114).

## Therapy

### Inhibition of the NF- κB signaling pathway activation

Spartolonin B can significantly inhibit toll-like receptor 4 (TLR4) and NF- κ B protein expression through the TLR4/MyD88/NF- κB pathway. In the rat lumbar model, Spartolonin B effectively reduced the histological score of disc degeneration and alleviated disc cell death (115). The selective NF- κ b-pathway inhibitor –JSH-23 can inhibit senescence and apoptosis of NPCs, as well as ECM degeneration induced by oxidative stress and inflammatory responses. The experiments showed that deficiency of ARG-2 produced similar results to JSH-23. Deficiency of ARG2, therefore, suppresses NF- κ B pathways to prevent DDD (116).

Additionally, the experiments showed that the expression of collagen 2 and aggrecan gradually decreased along with the expression levels of TFEB (Transcription factor EB) and QNZ (Quinazoline) as the degree of DDD increased. After over-expression of TFEB and QNZ, in NP cells, NF- κ B and ROS levels decreased, antioxidant enzymes upregulated, and inflammatory factors decreased. Studies have shown that both TFEB and QNZ inhibit inflammatory response and degradation of the ECM *via* NF- κ B signaling pathway, thereby slowing down degenerative changes of intervertebral discs (117, 118).

The findings showed that Celastrol decreased the expression levels of catabolic genes (MMP-3, 9, 13, ADAMTS-4, 5), oxidative stress factors (COX-2, iNOS), and pro-inflammatory factors (IL-6, TNF- α). Celastrol also inhibited NF- κ B pathways, thereby reducing I κ B α and phosphorylation of p65. Moreover, mangiferin antagonized the activation of the NF- κB signaling pathway induced by TNF- α, thereby reducing mitochondrial ROS activity and ameliorating mitochondrial damage. In conclusion, our study showed that celastrol and mangiferin could act on NF- κ B signaling pathway that reduces inflammatory factor-induced matrix catabolism and inflammatory response in NP cells and attenuates disc degeneration. In addition, Vitamin D and Naringin could inhibit the activation of the NF- κB signaling pathway *in vitro* and *in vivo* experiments, thereby suppressing the expression of p65 and Iκ B kinases α in NP cells (119–122).

### Inhibition of the ERK/MAPK pathway activation

AAPH induced oxidative stress and subsequent degenerative changes in NP cells *via* the ERK/MAPK pathway. PBN (N-tert-butyl-α-phenylnitrone) inhibited the activation of the ERK/MAPK pathway induced by AAPH(2,2’-azobis (2-amidinopropane) dihydrochloride) and attenuated the expression of matrix-degrading proteases and cell apoptosis. We preliminarily identified the underlying mechanism of the protective effect of PBN, which could protect IVD from oxidative stress damage and slow down the catabolism of cell-matrix and cell apoptosis. Additionally, Exenatide regulates ECM anabolic balance and restores disc degeneration by inhibiting MAPK activation and its downstream AP-1 activity. As potent drugs inhibiting the ERK/MAPK pathway, PBN and Exenatide provide therapeutic targets for the treatment of DDD (123, 124).

Both STS (Sodium tanshinone IIA sulfonate) and AQP-3 (aquaporin-3) inhibited the p38 MAPK pathway. In the experiment, STS could significantly reverse the lower level of Col2 and aggrecan as well as the higher level of MMP-3/13, IL-1β, IL-6, and TNF- α in the IDD group, and increased antioxidant enzyme activities, reducing the level of oxidative stress caused by acupuncture. AQP-3 could inhibit the expression of Bax and caspase-3 genes and alleviate NP cells’ apoptosis under oxidative damage. The inhibition of the p38 MAPK pathway by STS and AQP-3 has implications in further applications for alleviating the development of DDD (125, 126).

Additionally, we found that Que (Quercetin) inhibited the p38 MAPK signaling pathway through the p38 MAPK autophagy pathway. Que significantly reduced intracellular ROS levels by decreasing Bax and increasing Bcl-2 expression. Que also promotes ECM stability by increasing collagen II and aggrecan and decreasing MMP13 levels. Our results also showed that Que promoted the expression of autophagy markers Beclin-1, LC3-II/I and decreased p62. In a rat tail puncture-induced IVDD model, Que inhibited NPCs from undergoing apoptosis by regulating the p38 MAPK-mediated autophagy pathway (127).

*In vitro* studies have shown that Sirt3(sirtuin3) expression is reduced in degenerating NP tissue and that the activation of the AMPK/PGC-1 α pathway may partially alleviate NPC senescence caused by Sirt3 reduction. Therefore, the activation of AMPK/PGC-1α by up-regulating Sirt3 pathways delaying aging and NPC senescence is a potential therapeutic strategy for the treatment of IVDD (128).

### Inhibition of the P53-P21-RB/P16-RB pathway activation

Studies have shown that expression of Sirt1(sirtuin1) and Sirt2(sirtuin2) is significantly reduced in severely degenerated disc tissues. In degenerative disc disease, the inflammatory factor IL-1 β and ROS significantly contribute to DDD progression. However, the over-expression of Sirt1 and Sirt2 can reverse the IL-1 β effect and increase the production of the antioxidant SOD1/2. In addition, the p53 and p21 signaling pathways could be significantly inhibited by the over-expression of Sirt1 and Sirt2. These results suggest that Sirt1 and Sirt2 alleviate NP cell death by suppressing oxidative stress and inflammation *via* repression of the p53/p21 pathway. Sirt1 and Sirt2 can be novel targets for DDD therapy in the future (129).

### Restoration of mitochondrial function

Antioxidant administration and restoration of mitochondrial function may be as strategies for the treatment of IDD (122).BMSCs-exos were shown to dramatically decrease ROS and MDA levels, alleviating oxidative stress of NP cells. Meanwhile, mitochondrial membrane potential could be enhanced and the mitochondrial damage could be prevented by BMSCs-exos, then the proliferation and cytoactive of NP cells were stimulated (130).H2O2 and tert-butyl hydroperoxide (TBHP) were commonly used to stimulate the oxidative stress microenvironment. Bari et al. reported that MSCs-exos at the doses of 5 and 50 mg/ml could play a protective role for NP cells, against the H2O2-induced oxidative stress damage (131).In a mice model, platelet-rich plasma (PRP)-derived exosomal miR-141-3p suppressed the cytotoxicity of H2O2 on NP cells and slowed down senescence process of IVD through the activation of Keap1/Nrf2 pathway (132).Also, MSCs-derived exosomal miR-31-5p could inhibit the apoptosis and calcification of TBHB-induced endplate chondrocyte by reducing ATF6-related ER stress (133).

Mitochondria, including genome or other components, plays an important role in intercellular communication (134).Exosomes also participants in the process of mitochondrial transfer. MSCs-exos may deliver mitochondria-related proteins to NP cells and repair the mitochondrial malfunction (135).In a rabbit model, MSCs-derived exosome could preserve the proteoglycan in IVD and block the oxidative stress-induced ECM degradation (136).Additionally, owing to the inhibited formation of ROS and NLRP3 inflammasome by MSC-exos, the apoptosis of NPCs could be alleviated and the deterioration of IDD could be halted.

Humanin (HN), a mitochondria-related peptide translated by the open reading frame of mitochondrial 16S rRNA, plays a role in resisting cellular oxidative damage and inhibiting apoptosis in response to oxidative stress. Experimentally, exogenous HN was shown to reduce intracellular ROS content and the extent of cellular damage and to enhance cellular anabolism and mitochondrial function; this anti-oxidative effect was lost in a cellular model in which HN expression was inhibited (137).In treating age-related cataracts and macular degeneration, HN exerts a positive antioxidant effect. In oxidatively stressed human lens epithelial cells (HLECs) and retinal pigment epithelial cells (hRPE): HN protects cells by upregulating GSH expression levels in mitochondria, thereby resisting the impairment of mitochondrial function by oxidative stress and activating caspase 3 and caspase 4 expression (138, 139).

In ischemia-reperfusion injury, humanin (HN) and its analogs (HNG) also play an anti-oxidative stress role. HNG-treated rat cardiomyocytes (H9C2 cells) were placed in a hydrogen peroxide (H2O2) solution. The level of reactive oxygen species (ROS) expression in cells treated with HNG was significantly lower than in the control group. The structure, membrane potential, and ATP levels of mitochondria were maintained as usual (140).HNG induced activation of catalase and glutathione peroxidase (GPx) within 5 min and reduced the ratio of oxidized to reduced glutathione within 30 min. Red-eared turtles can withstand prolonged hypoxia without causing cellular damage in the presence of rapid oxygen reperfusion. In red-eared turtles, the presence of humanin homolog (TSE-humanin) was confirmed by ELISA and western immunoblotting. TSE-humanin induced catalase and glutathione peroxidase (GPx) activation to produce antioxidant effects in the presence of blood reperfusion (141).

Interestingly, humanin (HN) inhibits apoptosis and promotes the proliferation of growth plate chondrocytes. It was demonstrated that GCs-induced bone growth impairment and chondrocyte apoptosis were inhibited in bones of mice overexpressed with HN or treated with HN. Still, HN did not interfere with the expected anti-inflammatory effects of GCs (142). Further, HN protected chondrocytes even in inflammation (143). Altogether, these data from chondrocytes may also provide a new strategy for treating lumbar degenerative diseases.

### Polymeric biomaterials

The process of IDD could not be slowed down or reversed by the therapies that are now available. On the basis of this, polymeric biomaterials are now being developed, which have the potential to stimulate the regeneration and repair of the IVD. There were various advantages of polymeric biomaterials, including appropriate biomechanical characteristics and plasticity to restore the disc height and volume, and sustain the mechanical stress and segmental movement; excellent biocompatibility with no significant harmful effects on either autologous tissues or injected cells; good biodegradability, which indicated the ability to be metabolized and regenerated together with IVD (144, 145). These properties has brought forth fresh hope for the treatment of spinal degenerative illnesses.

Numerous experiments have confirmed that polymeric biomaterial therapy is essential for intervertebral disc repair at the molecular and cellular levels. Clinical investigations have demonstrated the ability of polymer-peptide hydrogels to promote the transformation of adult myeloid cells from a degenerative fibroblast-like state to a juvenile myeloid phenotype. *In vitro* three-dimensional culture, encapsulation of adult degenerative NP cells in a rigid formulation of hydrogels with laminin-mimetic peptides IKVAV and AG73 promotes the biosynthetic capacity of NP cells. In animal experiments, rabbit NP cells were inoculated on biodegradable nanofiber (NF) scaffolds and regenerated NP-like tissues *in vitro* and a subcutaneous implantation model. Extracellular matrix (ECM) (glycosaminoglycan and type II collagen) production was significantly higher on NF scaffolds than on control SW scaffolds. ASC-loaded collagen hydrogels were additionally implanted into the degenerated lumbar spine tissue, and the height of the degenerated discs was analyzed at 6 and 12 months of implantation. The size of the degenerated disc remained stable, without significant loss, but did not fully recover. As a therapeutic strategy to address degenerative disc degeneration, polymeric biomaterial therapy can deliver nutrients, growth-promoting factors, and active cells to the degenerative disc, effectively combating lumbar disc degeneration and lumbar spine cellular aging.

Additionally, the role of antioxidant was also found in some polymeric biomaterials. Chitosan is a naturally occurring amino polysaccharide that is produced by the deacetylation of chitin. Chitin is the precursor to chitosan. Research on tissue engineering has made extensive use of chitosan because of its low cytotoxicity, excellent biocompatibility, *in-vivo* biodegradability, and antibacterial characteristics (146). Chitosan hydrogels could be as carriers of BMSCs, fibroblast growth factor, or diclofenac, which indeed improve the versatility of this polymeric biomaterial, including antioxidant effects (147).

Hyaluronic acid (HA) is a polymer that is made up of D-glucuronic acid and N-acetylglucosamine. It is a water-soluble polysaccharide that is often found in the epithelium and connective tissue of animals. Hyaluronic acid is also the principal component of the extracellular matrix in humans (ECM) (148). Additionally, HA has beneficial characteristics as an anti-inflammatory and an antioxidant. This effect was favorable to IDD that was followed by local oxidative stress and was exerted by high molecular weight HA *via* the classical IFN- signaling pathway, which was reported by Kazezian et al. (149).

The issue of immunogenicity may be sidestepped by using fibrin gel since it is already present in blood. It is advantageous in that it consists of basic materials, can be prepared quickly, has a reasonable level of toughness, and has good application prospects in the field of IVD disc tissue engineering (150). Silk fibroin (SF) is a natural high-molecular-weight fibre protein that is derived from silk. It has the same 18 amino acids that are found in the human body (151). The β-folding structure that is present in the crystallization region of the protein is associated with the greatly increased mechanical capabilities that SF hydrogels display (145). Bari et al. produced a microglue using a combination of silk protein, platelet-poor plasma, and regenerated platelet lysate. The IVD could be protected against apoptosis attributed to the anti-oxidant properties of the composite hydrogel, which played a role in cleaning the reactive oxygen species in IVD while also controlling the oxidative stress process and reducing inflammation. The properties and therapeutic effects of several therapies were summarized in **Table 2** (152).

**Table 2:** Therapeutic effects of therapeutic drugs on intervertebral disc cellular senescence.

Drugs	Drug properties	Therapeutic effects
Spartolonin B	Antagonist of both TLR2 and TLR4	Inhibited the expression of the Toll Like Receptor 4 (TLR4) and NF-KB
JSH-23	Inhibitor of NF-κB	Inhibited senescence, apoptosis and ECM degeneration
TFEB	A transcription factor	Inhibited inflammatory response and degradation of extracellular matrix
QNZ	Inhibitor of NF-κB	Inhibited inflammatory response and degradation of extracellular matrix
Celastrol	Inhibitor of proteasome	Downregulates the expression of catabolic genes, oxidative stress factor and pro-inflammatory factors
Vitamin D	Fat soluble vitamins	Inhibition of the NF- κB signalling pathway activation
Naringin	Inhibitor of NF-κB	Inhibition of the NF- κB signalling pathway activation
Mangiferin	Nrf2 activator and inhibitor of NF-κB	Inhibition of the NF- κB signalling pathway activation
PBN	A free-radical scavenger	Inhibition of the ERK/MAPK pathway activation Downregulates the expression of matrix degrading proteases
Exenatide	A glucagon-like peptide-1 receptor agonist	Inhibition of the MAPK pathway activation and AP-1 activation.
STS	A water-soluble derivate of tanshinone IIA (Tan IIA)	Upregulation the expression of Col2 and aggrecan Promoted the activation of antioxidant enzyme
AQP-3	An intrinsic membrane protein	Downregulates the expression of Bax and caspase-3 gene
Que	A natural flavonoid	Upregulation the expression of Bcl-2 aggrecan and type II collagen Downregulates the expression of Bax, MMP-3 and MMP-13
SIRT3	A deacetylase class	Promoted the activation of AMPK/PGC-1α
Sirt1 and Sirt2	A deacetylase class	Suppressing oxidative stress and inflammation
BMSCs-exos	Small membrane vesicles containing complex RNAs and proteins	Scavenging of intracellular reactive oxygen species Recovery of mitochondrial function Inhibition of apoptosis Promotes cell proliferation and differentiation
MSCs-exos	Small membrane vesicles containing complex RNAs and proteins	Recovery of mitochondrial function Inhibits degradation of extracellular matrix Inhibition of apoptosis Inhibition of calcification at the EP Promotes cell proliferation and differentiation
Humanin (HN)	mitochondria-related peptide	Reducing intracellular ROS content Enhanced cellular anabolism Recovery of mitochondrial function Inhibition of apoptosis
Polymeric Biomaterials(PB)	Biopolymers	Induce regeneration and repair of IVD.
Hyaluronic acid(HA)	water-soluble polysaccharide	Controls and alleviates oxidative stress processes and inflammatory responses
Silk Fibroin (SF)	natural high-molecular-weight fibre protein	Removes the active oxygen species aspect in the IVD. Controls and alleviates oxidative stress processes and inflammatory responses

### Conclusion and future directions

IDD is a significant risk factor causing low back pain and nerve compression in the lower extremities, which imposes a heavy economic burden on patients and society. With aging and in the role of multiple related factors, ROS accumulate in the IVD, leading to senescence and apoptosis of the disc cells through various mechanisms of action and eventually leading to IDD. The main pathways and targets of oxidative stress that inhibit normal cell proliferation and differentiation and induce cellular senescence and apoptosis remain to be verified. However, based on the current research on the mechanism of action, we summarized four signaling pathways and related cytokines for oxidative stress-induced senescence and apoptosis in IVD cells. Oxidative stress regulates the transcription and translation of downstream target genes through the aforementioned cellular signaling pathways and cytokines, leading to pathological changes such as the release of inflammatory factors, methylation of DNA structures, and cellular autophagy. Antioxidant therapy is considered the most promising therapeutic tool for the treatment and prevention of IDD. This article presents preclinical and clinical therapeutic strategies against oxidative stress to find therapeutic drug targets for the different molecular mechanisms of oxidative stress-induced IDD so that more effective and safer drugs can be applied to prevent and treat IDD. We still need to track cutting-edge basic research experiments and clinical trials to develop new therapeutic agents for the treatment and prevention of IDD and mitigate the burden on patients and society.

## Author contributions

HY, YC, and YZ contributed to conception and design of the study. FC, HY, and YC collected the documents and wrote the first draft of the manuscript. YZ wrote sections of the manuscript. FC, HY and YZ designed the figures. HY, YC, YL, YH, and YZ critically revised the manuscript. YL, YH, and YZ supervised the manuscript. All authors have read and approved the submitted version.
